# Specific capture and whole-genome phylogeography of Dolphin morbillivirus

**DOI:** 10.1038/s41598-020-77835-z

**Published:** 2020-11-30

**Authors:** Francesco Cerutti, Federica Giorda, Carla Grattarola, Walter Mignone, Chiara Beltramo, Nicolas Keck, Alessio Lorusso, Gabriella Di Francesco, Ludovica Di Renzo, Giovanni Di Guardo, Mariella Goria, Loretta Masoero, Pier Luigi Acutis, Cristina Casalone, Simone Peletto

**Affiliations:** 1 Istituto Zooprofilattico Sperimentale del Piemonte, Liguria e Valle d’Aosta, Torino, Italy; 2 Atlantic Center for Cetacean Research, University Institute of Animal Health and Food Safety (IUSA), Veterinary School, University of Las Palmas de Gran Canaria, Canary Islands, Spain; 3 Laboratoire Départemental Vétérinaire de l’Hérault, Montpellier, France; 4 Istituto Zooprofilattico Sperimentale dell’Abruzzo e del Molise “G. Caporale”, Teramo, Italy; 5 Faculty of Veterinary Medicine, University of Teramo, Teramo, Italy

**Keywords:** Phylogenetics, Metagenomics, Viral epidemiology, Viral evolution

## Abstract

Dolphin morbillivirus (DMV) is considered an emerging threat having caused several epidemics worldwide. Only few DMV genomes are publicly available. Here, we report the use of target enrichment directly from cetacean tissues to obtain novel DMV genome sequences, with sequence comparison and phylodynamic analysis. RNA from 15 tissue samples of cetaceans stranded along the Italian and French coasts (2008–2017) was purified and processed using custom probes (by bait hybridization) for target enrichment and sequenced on Illumina MiSeq. Data were mapped against the reference genome, and the novel sequences were aligned to the available genome sequences. The alignment was then used for phylogenetic and phylogeographic analysis using MrBayes and BEAST. We herein report that target enrichment by specific capture may be a successful strategy for whole-genome sequencing of DMV directly from field samples. By this strategy, 14 complete and one partially complete genomes were obtained, with reads mapping to the virus up to 98% and coverage up to 7800X. The phylogenetic tree well discriminated the Mediterranean and the NE-Atlantic strains, circulating in the Mediterranean Sea and causing two different epidemics (2008–2015 and 2014–2017, respectively), with a limited time overlap of the two strains, sharing a common ancestor approximately in 1998.

## Introduction

Cetacean morbillivirus (CeMV) is a member of the genus *Morbillivirus* (family *Paramyxoviridae*, subfamily *Orthoparamyxovirinae*), which includes also the Canine morbillivirus, Feline morbillivirus, Measles morbillivirus, Phocine morbillivirus, Rinderpest morbillivirus, and Small ruminant morbillivirus^1^. CeMV is able to infect a wide range of host species, and five different subspecies have been described so far: CeMV-1, CeMV-2, CeMV-3, CeMV-4, and CeMV-5, corresponding to Dolphin morbillivirus (DMV), Porpoise morbillivirus, the Pilot Whale morbillivirus, the Longman’s Beaked Whale morbillivirus and Guiana Dolphin morbillivirus^2–5^. DMV caused several outbreaks worldwide in the last decades, with those of 1990–1992 and 2006–2008 in the Western Mediterranean and with that of 2013–2014 along the Atlantic USA coastline being the most dramatic ones and involving different species^6–8^. Dolphin morbillivirus (DMV), similarly to many other animal and human morbilliviruses, is a primary lymphotropic, epitheliotropic and neurotropic pathogen, which is shed outside infected cetacean hosts via the respiratory, ocular, fecal and urinary routes. Virus-carrying aerosols and oculo-conjunctival excreta/secreta are believed to be of particular concern, based upon the gregarious social behaviour of many cetacean species, which facilitates viral transmission. Furthermore, it has also been reported that DMV-infected dolphins may carry the virus across 220–230 km marine distances, thereby remaining infectious throughout a 24 days-long period^8^. Apparently, each outbreak is caused by a novel virus strain that finds a naïve cetacean population due to a decrease of the herd immunity^5^. Actually, it was estimated that seropositivity against morbilliviruses in mature dolphins decreased from 100% in 1990–1992 to 50% in 1997–1999, although with a small sample size^9^. A similar framework was observed in the East and Gulf of Mexico coasts of the United States^10^.

The genome of CeMV is a negative sense non-segmented single-stranded RNA, with a length of 15,702 bp, containing six open reading frames (N, P/V/C/, M, F, H and L) that encode for eight proteins^11^. For more than 10 years, only one CeMV genome sequence was available. Since 2018 additional sequences have been published, obtained with three different approaches, including multiple PCR followed by Sanger sequencing and NGS starting from either cultured isolates or tissue samples^3,12,13^.

Direct sequencing of a virus from tissue may be considered the best approach, although it requires a very high-throughput as reads mapping to the virus are usually very low, even < 1%, and the complete sequence may not be retrieved^3,14^. Several methods to enrich the viral RNA and to increase its yield have been described, including filtration, centrifugation, nuclease treatment before RNA purification, but results do not fully agree on a successful outcome. Some Authors suggest not to enrich the sample to avoid loss of viral RNA^15^, while others obtained a better outcome with three treatments before purification^14^. Virus isolation may be an easy enrichment step, but not all the viruses can be isolated on cell cultures, and adaptation may induce nucleotide modifications. In our previous work, we successfully isolated DMV using Vero.DogSLAMtag cells, and Jo and colleagues isolated two strains, although viral isolation is not guaranteed^3,13^. Targeted enrichment of RNA or DNA of interest using custom baits is a well described procedure in gene expression profile studies, but its application is still limited in virology. For example, this approach was used to retrieve Norovirus genomes, whose sequencing is challenging due to viral genome heterogeneity, with their isolation on cell culture being troublesome^16^.

In this study, we successfully generated 15 DMV whole-genome sequences from total RNA using the Agilent Technologies SureSelect XT RNA Direct kit with DMV-specific bait probes designed on available genomes. Furthermore, we compared the obtained sequences with those publicly available and performed a phylogenetic and phylodynamic analysis in order to describe the evolution and spatio-temporal distribution of DMV in the Mediterranean Sea.

## Results

### RNA purification, quality check, library preparation and sequencing

Among the tissue samples tested by real-time RT-PCR, 15 were selected based on the collection site and year in order to increase the spatio-temporal coverage of DMV strains (Table 1). The SureSelect XT RNA Direct kit requires different input amount of RNA based on starting RNA size. To assess this parameter, we analyzed the 15 RNA samples with a small-scale gel electrophoresis on a RNA 6000 Nano chip. The RNA Integrity Number (RIN) of the candidates for sequencing, when achieved, was between 1.70 and 5.30 (data not shown). These values indicate a high degradation of the total RNA, thus the poor FFPE RNA protocol was adopted.

**Table 1:** Information on samples with details about host species, body tissues, stranding location and year.

Animal ID	Body tissues	Species	Year	Collection site	Sea	Mapping reads (raw count)	Mapping reads (%)	Total read number	Average coverage
59,728	Mediastinal lymph node	*Balaenoptera physalus*	2011	Italy (SS)	Pelagos Sanctuary	746,745	93.9	795,283	4082
19,929	Heart	*Tursiops truncatus*	2013	Italy (ME)	Tyrrhenian	446,892	77.2	578,805	1864
3908	CNS	*Stenella coeruleoalba*	2015	Italy (IM)	Pelagos Sanctuary	14,457	0.7	2,042,677	99
80,729	CNS	*Stenella coeruleoalba*	2011	Italy (SA)	Tyrrhenian	1,807,114	96.1	1,880,853	4293
85,537	Spleen	*Stenella coeruleoalba*	2010	France	Pelagos Sanctuary	12,986,524	98.4	13,194,219	7822
85,548	Lung	*Stenella coeruleoalba*	2008	France	Pelagos Sanctuary	2,339,785	97.4	2,402,228	6883
59,780	Spleen	*Physeter macrocephalus*	2014	Italy (CH)	Adriatic	21,650	8.4	257,542	137
92,300	Lung	*Stenella coeruleoalba*	2016	Italy (LE)	Adriatic	14,674,119	98.1	14,958,468	7819
95,842	CNS	*Stenella coeruleoalba*	2016	Italy (GE)	Pelagos Sanctuary	203,667	26.5	767,178	1856
78,983	CNS	*Stenella coeruleoalba*	2017	Italy (SV)	Pelagos Sanctuary	61,609	10.2	605,659	389
6023	Lung	*Stenella coeruleoalba*	2017	Italy (LE)	Adriatic	5,230,337	97.7	5,354,356	7640
6020	Skeletal muscle	*Stenella coeruleoalba*	2017	Italy (TA)	Adriatic	67,107	15.5	431,804	427
26,823	CNS	*Stenella coeruleoalba*	2017	Italy (IM)	Pelagos Sanctuary	32,581	4.7	689,559	207
3618	CNS	*Stenella coeruleoalba*	2009	Italy (RM)	Tyrrhenian	3,034	0.5	657,260	30
123,517	Cell culture from CNS	*Stenella coeruleoalba*	2008	Italy (SV)	Pelagos Sanctuary	825,625	93.3	885,034	3292
22,497	CNS	*Stenella coeruleoalba*	2017	Italy (PE)	Adriatic	–	–	–	–
20,673	Heart	*Stenella coeruleoalba*	2016	Italy (LE)	Adriatic	–	–	–	–

For Italian samples, the province abbreviation is reported: SS, Sassari; ME, Messina; IM, Imperia; SA, Salerno; CH, Chieti; LE, Lecce; GE, Genoa; SV, Savona; TA, Taranto; RM, Rome; PE, Pescara. Data about sequencing are also reported for samples processed with target enrichment. For sequencing, output of the sequencing sessions in terms of reads mapping to the viral genome, total read number, and average coverage are indicated. Sequencing data of samples processed with SISPA method are not reported.

The quantification of the libraries indicated that the protocol was successfully performed, although sample 3618 had a DNA concentration lower than the required input, and it was sequenced separately in a second run.

### Data analysis and phylogenetics

After quality trimming, the total read number was 46,095,682 (mean: 2,880,980; min: 257,542; max: 14,958,468). The genomes were sequenced with a very high average coverage, > 100X for most of the samples (Table 1). The proportion of reads mapping to the viral genome was very high, > 90% for seven samples, and even in those ones with a lower percentage of enrichment (< 30% reads mapping viral genome), the final coverage was satisfying. Only sample 3618 had a low coverage, which led to a partially complete genome.

Other than DMV, in some cases we also detected sequences of potentially pathogenic bacteria: *Photobacterium damselae* (n = 6; 80,729, 85,537, 85,548, 59,780, 105,168, 92,300, 95,842, 78,983, 6023, 6020); *Clostridium* spp. (n = 4; 19,929, 3908, 80,729, 85,537, 85,548, 59,780); *Mannheimia haemolytica* (n = 1; 85,537); *Salmonella enterica* (n = 1; 26,823).

Nucleotide sequence identity for the whole genome was > 98% among all the aligned sequences. More in detail, three sequences showed a lower similarity: the reference genome NC_005283 (98.9% average similarity) and two North Sea samples MH430940 and MH430941 (98.5% and 98.7%, respectively, compared to all the other sequences). A similarity matrix is reported in Table S1. The sliding window analysis, comparing the sequence identity with 20 bp steps, highlighted a region in the L gene of the reference genome NC_005283, with a length between 12.000 and 12.500 nt, exhibiting a much lower similarity (up to 90%) than the other DMV gene sequences (Fig. S1). A recombination analysis on the studied genomic sequences detected four recombination events, two of which in the reference sequence NC_005283 (Table S2). In details, several methods detected a recombination event at 90-10440nt, with the major parents being most likely the other contemporary strains from Balearic, 1990 (MH430934-16A and MH430935-muc), and another at 12,091–12,459 nt, with the major parent being sample 3618. A third recombination event involved the sequences MH430937, KU720623, and again sample 3618 with the first sequence as probable recombinant. The last recombination event was detected in sequence MH430939, with samples 22,497 or 78,983 as major parental sequences. In order to test if the recombination events on the reference genome affected the phylogenetic reconstruction, two different trees were built with MrBayes using the alignment after removing the NC_005283_ES_Balearic_1990 sequence or the MH430934-16A_ES_Balearic_1990 and MH430935-muc_ES_Balearic_1990 ones (data not shown). The topology of the obtained trees did not differ dramatically, but we preferred to remove the reference genome NC_005283 for all the phylogenetic analyses.

**Figure 1:**
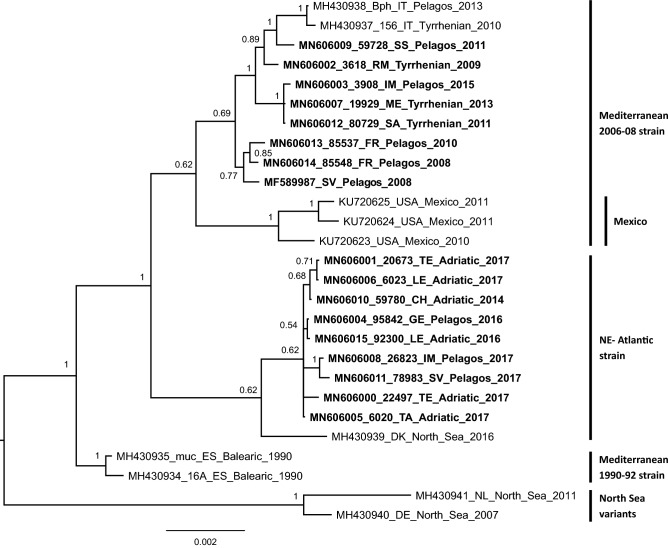
Bayesian phylogenetic tree based on the nucleotide genome sequence of DMV. The tree was inferred by MrBayes using the genomic data partitioned by gene, with the proper nucleotide substitution model, according to the bModelTest results: GTR for H, M, and PVC genes, GTR + G + I for F, N, and L genes, and JC69 + G + I for the noncoding partition. Taxa names in bold are the new sequences presented in this work. For Italian samples presented here, the province abbreviation is reported in the taxon name: SS: Sassari; ME: Messina; IM: Imperia; SA: Salerno; CH: Chieti; LE: Lecce; GE: Genoa; SV: Savona; RM: Roma. Strains mentioned in the manuscript are reported close to the related clade. Statistical support to the internal nodes is reported as posterior probability (pp).

The Bayesian phylogenetic tree (Fig. 1) well discriminated the NE-Atlantic and the Mediterranean strains, with samples from the Gulf of Mexico forming a separate clade within the Mediterranean strain, confirming previous results^12^. The NE-Atlantic strain was composed mainly by our samples from the Mediterranean Sea and a sample from North Sea (Denmark, 2016). As already reported, two samples collected in the North Sea from white-beaked dolphins (*Lagenorhynchus albirostris*) in 2007 and 2011, formed a well separated clade, named the North Sea variant (NSV)^17^. Another interesting point about the two strains circulating in the Mediterranean Sea is their temporal separation with a partial overlap, since the Mediterranean strain was detected from 2008 to 2015 and the NE-Atlantic strain from 2014 to 2017.

A detailed analysis on the translated sequences was performed to identify amino acid substitutions that might be specific to a given strain, thus single mutations in a few samples were omitted (Table 2). Also, the noncoding region was excluded from this analysis. The considered strains were: Mediterranean including Mexico, Mexico, NE-Atlantic, and NSV. The majority of the substitutions were detected in the NSV strain, compared to all the other sequences, with 22 non-synonymous substitutions, 11 conservative and 11 non-conservative. In the Mexican strain, a total of nine substitutions were observed, of which only two non-conservative, in H (D312N) and in L genes (R2016I). Seven sites had a different amino acid between the Mediterranean and the NE-Atlantic strains, with two of them being non-conservative replacements. Interestingly, at these positions, the high-throughput sequencing could detect both variants in four samples (3908, 6020, 95,842, 59,780). Strain-specific amino acid changes on L gene were observed only in NSV and Mexican samples. Sample 3618 was analyzed singularly to detect possible substitutions that may explain a different morbilliviral disease phenotype responsible for BOFDI, and only two were reported: R509H in the N gene, and D425B in the F gene, which is a partial substitution, being B either Aspartate (D) or Asparagine (N). A protein 3D structure prediction with PSISPRED-Bioserf and PHYRE2 was assessed to test whether the R509H may change the folding, but no difference was detected (data not shown)^18,19^.

**Table 2:** Amino acid substitutions between the different DMV strains under study.

Gene	Mediterranean/NE-Atlantic		North Sea Variants		Mexico		BOFDI (ID 3618)	
	Substitution	Type	Substitution	Type	Substitution	Type	Substitution	Type
**M**	–	–	I201V	Conservative	–	–	–	–
	–	–	R225C	Non-conservative	–	–	–	–
**N**	I21T	Non-conservative	A135T	Non-conservative	K495R	Conservative	R509H	Non-conservative
	N505S	Conservative	–	–	–	–	–	–
**H**	A164V	Conservative	E161D	Conservative	V220I	Conservative	–	–
	A499V	Conservative	E169G	Non-conservative	D312N	Non-conservative	–	–
	–	–	F233L	Conservative	–	–	–	–
	–	–	K371R	Conservative	–	–	–	–
	–	–	Q502R	Non-conservative	–	–	–	–
	–	–	I598L	Conservative	–	–	–	–
**F**	P10L	Conservative	K284R	Conservative	R538K	Conservative	D425B	Non-/conservative
	–	–	–	–	R540K	Conservative	–	–
**L**	–	–	D93A	Non-conservative	G1972V	Conservative	–	–
	–	–	A154V	Conservative	R2016I	Non-conservative	–	–
	–	–	T185I	Non-conservative	–	–	–	–
	–	–	G639S	Non-conservative	–	–	–	–
	–	–	V1006L	Conservative	–	–	–	–
	–	–	I1016V	Conservative	–	–	–	–
	–	–	I1343V	Conservative	–	–	–	–
	–	–	S1627P	Non-conservative	–	–	–	–
	–	–	I1696T	Non-conservative	–	–	–	–
	–	–	I1867V	Conservative	–	–	–	–
	–	–	I2107N	Non-conservative	–	–	–	–
**PVC**	P105S	Non-conservative	P223S	Non-conservative	L58P	Conservative	–	–
	V421L	Conservative	–	–	K489R	Conservative	–	–

Substitutions were reported if present in all the sequences of a given strain: Mediterranean vs. NE-Atlantic, North Sea Variants, Mexico, and those unique for the BOFDI specimen (ID 3618).

**Figure 2:**
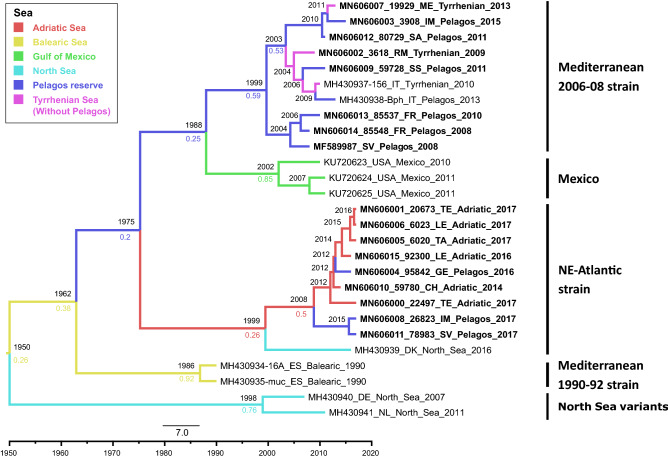
Maximum Clade Credibility phylogeny based on the whole genome sequence of DMV. The phylogeny was inferred from the complete genome sequences partitioned by gene and noncoding region. Tip dates and collection site, reported as sea of origin, were included in the MCMC analysis. Branches are colored based on the most probable location. At internal nodes, the estimated tMRCA is reported. Strains are shown on the right side. The probability for the most probable location is reported for the most relevant nodes, colored according to the sea.

The phylogeographic tree from the BEAST analysis (Fig. 2) confirmed the topology obtained with MrBayes, although the clade of the NE-Atlantic strain had a higher posterior probability (pp) and it was more resolved in the former than the latter. The phylodynamic inference estimated the geographical origin of DMV with a strong posterior probability only in the internal nodes within a clade. Within the NE-Atlantic clade, the most probable origin was estimated from the Adriatic Sea, with a weak support (pp = 0.5) for the internal nodes. Within the Mediterranean clade, the most probable area of origin for the Mediterranean samples was the Pelagos Sanctuary (pp = 0.59), and Mexico for the Mexican samples (pp = 0.85). However, the common ancestor had a weak probability (pp = 0.25) of being placed in the Pelagos. A densitree plot of the posterior probability for the location graphically showed the uncertainty in some of the internal nodes (Fig. S2).

Based on the molecular clock, the tMRCA was estimated to 1999 (95% HPD Interval: 1966–2003) for the Mediterranean strain, to 2002 (95% HPD Interval: 1993–2008) for the Mexican clade, to 1999 (95% HPD Interval: 1984–2011) for the NE-Atlantic strain, and to 1986 (95% HPD Interval: 1982–1989) for the clade of the 1990–1992 strain, whle the tMRCA at the root was estimated to 1950 (95% HPD Interval: 1896–1986). The common ancestor of the Italian samples within the NE-Atlantic strain was estimated to be in 2008 (95% HPD Interval: 2002–2013), when it evolved into two separate variants, one mostly circulating in the Adriatic Sea and the other one circulating in the Pelagos Sanctuary.

## Discussion

DMV has been the causative agent of a series of outbreaks, causing mass strandings and die-offs among free-ranging cetaceans in the Western Mediterranean Sea. Since its first detection in 1990, only a limited number of genome sequences have been displayed in public databases, leading to a limited knowledge about its evolutionary history. In the present study, we selected 15 DMV positive samples from tissue banks plus a cell culture isolate, and applied a targeted enrichment with custom baits to sequence the full viral genomes. Although the RNA integrity was very low, we successfully obtained an enrichment from our starting material, adopting the protocol for highly degraded FFPE-derived RNA. All the animals analyzed in the study were found stranded on the beach, almost always they were dead or dying condition. This, of course, had a strong impact on the quality of RNA purified from their body tissues.

In 7 out of 16 samples, we obtained > 90% viral reads, which allowed a very high coverage. Moreover, we obtained a good coverage even with a reduced output and lower percentage of viral reads. For example, from sample 59,780, we obtained only 257,542 reads, and only 8.4% (21,650 reads) mapped to the DMV genome. However, the average coverage of the contig was as high as 137X. Only for sample 3618 we could not retrieve the whole viral genome sequence, and we had a few scaffold regions.

Compared to a PCR amplification and Sanger sequencing approach, target enrichment may be more expensive, although Sanger cannot provide information about nucleotide variants. A PCR pre-amplification step can be also used before NGS library preparation, designing primers for long-distance PCR of 2000–5000 bp, as reported for Bovine Viral Diarrhea virus and West Nile virus^20,21^, although they might introduce amplification bias and need to be conserved between different strains. Moreover, from our experience, the amplification of N, PVC and F genes for typing is not always successful in positive samples. Thus, a pre-amplification using long PCR assays for NGS sequencing may be an issue of concern for some samples. Direct NGS sequencing of total RNA, as reported earlier, has the great disadvantage of generating a huge amount of data when only less than 1% is desired. An additional drawback of this massive approach may be also the generation of an incomplete genome sequence of the virus of interest, as reported by Jo and colleagues^3^, and also in the case of sample 22,497, generated by a traditional metagenomic approach. Of course, the metagenomic approach is more exhaustive if the aim of a study is not a single virus, but instead the whole microbiota. However, even with a target enrichment, we were able to identify some potentially pathogenic bacteria like *P. damselae*, *S. enterica*, and *Clostridium* spp., which were already reported in stranded cetacean specimens in the Mediterranean Sea^22–24^.

The comparison between genomic sequences showed that the virus, although with an evolutionary rate of 1.44 × 10^–4^ (95% HPD Interval: 5.14 × 10^–5^, 2.39 × 10^–4^) nucleotide substitution/site/year, is well conserved, with a sequence identity > 98%. The peak of dissimilarity of the reference genome in the L gene was first observed by Peletto and colleagues, and was explained as a strain-derived difference, based on two sequences only. Subsequently, Jo and colleagues compared the reference genome with other two of the same outbreak and suggested that this difference may be due to sequencing artifacts in the reference genome, and excluded the L gene from the phylogenetic analyses. Based on our novel data, we detected a recombination event occurring in the region with low similarity, that explains such large distance in a gene that, coding for the polymerase, is usually the most conserved gene in viruses. For this reason, we excluded the reference genome from the BEAST analysis.

Apparently, the only BOFDI case herein investigated was not caused by a specific DMV genotype, differently from what reported in Measles morbillivirus-infected humans with subacute sclerosing panencephalitis, in which a well-defined molecular signature has been reported within the M viral gene^25^. In this respect, since no peculiar amino acid substitutions were found within the DMV genome from the aforementioned BOFDI-affected striped dolphin^26^, other extrinsic causes should be investigated, along with host-derived factors driving prolonged viral persistence inside the CNS from dolphins affected by this intriguing neurologic disease form^27^.

The identification of a pattern of amino acid changes between different DMV strains may be relevant for both diagnostic and typing purposes. Specific assays could be designed to detect differences at a single nucleotide level to identify a strain with the aim of understanding the epidemiology of DMV infection, similarly to some previous assays^28^. The phylogenetic analysis could also be used to differentiate viral isolates. In fact, we were able to characterize some of the different strains that circulated in the last 30 years in the Mediterranean basin. As observed for other morbilliviruses, different strains follow each other throughout time, and probably we witnessed the NE-Atlantic strain replacing the Mediterranean 2006–2008 one. In fact, samples belonging to the Mediterranean 2006–2008 strain were collected between 2008 and 2013, and only one in 2015; conversely, samples belonging to the NE-Atlantic strain were collected in the Mediterranean basin after 2016, with only one in 2014. The NE-Atlantic strain was detected in 16 cetaceans stranded in 2011–2013 on the Galician and Portuguese shores^29^. Unfortunately, data about DMV strains in the Mediterranean Sea from the years 2014–2015 are lacking. An hypothesis may be that the NE-Atlantic strain was introduced in the Mediterranean Sea in 2014 by the Strait of Gibraltar, known to be an area of contact between cetaceans from the two seas. The pod that was found stranded on the Adriatic shores of Italy^3,30,31^ may have come into contact with a carrier pod from the Atlantic Ocean^32^. Either *Globicephala melas* and *Stenella coeruleoalba* may have introduced of the NE-Atlantic strain to the naive cetacean population of the Mediterranean Sea, due to their well documented roles as DMV carriers^8,33^. Thus introduced, the “novel” lineage completely replaced the previous one by the end of 2015 because the population was naïf to this virus and did not have immunity against the new variant. Phylogeography indicated the Adriatic Sea as the most probable location for the MRCA of the NE-Atlantic strain, although geographical information was limited. Furthermore, the mass stranding may be a hint of a novel virus variant infecting a naive population. Interestingly, four samples presented amino acid polymorphisms at positions that characterize the Mediterranean and the NE-Atlantic strains. This may be a clue for a co-infection by the two strains within the same host, but more data are required to better explain this phenomenon. According to our results, DMV was initially located in the Balearic Sea but this has a weak posterior probability, and it may be strongly influenced by the limited number of samples from the 1990–1992 outbreak, which were all collected in this area. Opposite to previous estimates, the isolate DMV/DK/2016 does not share the same MRCA with the Mexican samples, and instead it is included in the NE-Atlantic clade, although other genomic sequences were not available^12^. In our study, we generated nine novel sequences that filled this gap, contributing to better estimate the divergence times.

Under the “one health” framework, a wider perspective of CeMV is becoming more and more important, due to its progressively expanding host range in the Mediterranean Sea. As previously described, morbilliviruses are prone to host species jumps, like CeMV infecting several species of marine mammals, of canine distemper virus, able to infect non-human primates^32,34^. In conclusion, genomic information on the DMV strain of Cetacean morbillivirus are essential to understand its evolution as well as to infer its spatio-temporal trends also in the past, since the only available information relies on stranded animals. In the future, more sequences will be probably available, so that better estimates will be also obtained.

## Methods

### Sample collection and selection

Samples were selected from those preserved in the tissue banks of C.Re.Di.Ma (Centro di Referenza Nazionale per le Indagini Diagnostiche sui Mammiferi marini spiaggiati, Italian National Reference Center for diagnostic activities in stranded marine mammals), of the Università degli Studi di Padova, and of the Laboratoire Départemental Vétérinaire de l’Hérault (Montpellier, France).

A collection of 23 samples from DMV-infected cetaceans, found stranded dead between 2008 and 2017 on the Italian and French shores were selected to confirm the presence of viral RNA by real time PCR. Among them, 13 were selected in order to cover the major outbreaks in the Mediterranean Sea. One sample (ID 3618) from a striped dolphin affected by a "brain-only" form of DMV infection (BOFDI) was kindly provided by Prof. Giovanni Di Guardo, Università degli Studi di Teramo, and Dr. Cristiano Cocumelli, Istituto Zooprofilattico Sperimentale del Lazio e Toscana^27^. Finally, we included the RNA from the previously isolated strain DMV_IZSPLV_2008, as a positive control for target enrichment and sequencing^13^.

Two more tissue samples of striped dolphins (ID 22,497 and 20,673) were included in the study and processed with traditional metagenomics approach by Istituto Zooprofilattico Sperimentale dell’Abruzzo e del Molise (Dr. Alessio Lorusso and Dr. Gabriella Di Francesco). Sample ID 20,673 was kindly provided by Dr. Antonio Parisi (Istituto Zooprofilattico Sperimentale della Puglia e della Basilicata).

No live animals were used for this research.

### RNA purification and quality check

RNA purification was performed with the TRI Reagent (Sigma-Aldrich S.r.l., Milan, Italy) starting from 100 mg of tissue, with a final elution in 50 µl of nuclease-free water. RNA yield and purity (260/280 and 260/230 absorbance ratios) were assessed by spectrophotometer (VivaSpec LS, Sartorius, Goettingen, Germany), and yield was estimated more precisely with Qubit RNA HS Assay kit. Integrity was evaluated with the Agilent RNA 6000 Nano Kit (Agilent Technologies, Santa Clara, CA, USA) on the BioAnalyzer 2100.

The presence and the titer of viral RNA were evaluated by a semi-quantitative real time RT-PCR with two primer pairs: CeMV-He1/CeMV-He2, targeting 232 bp of the H gene, and DMVFu-F/DMVFu-R, targeting 191 bp of the F gene^35^. The reaction mix was prepared according to the QuantiFast SYBR Green RT-PCR Kit (Qiagen, Hilden, Germany) manual: 12.5 µl 2 × QuantiFast SYBR Green RT-PCR Master Mix, 1 μM of each primer, 0.25 μl QuantiFast RT Mix, 4.75 µl H_2_0, and 5 µl RNA, and run on a Stratagene Mx3005P instrument (Thermo Fisher, Waltham, MA, USA). Samples with Cq ≤ 30 were considered adequate for further processing.

### Probe design, library preparation and sequencing

Total RNA was processed for target enrichment by the SureSelect XT RNA Direct kit (Agilent Technologies), according to the manufacturer’s protocol, using 200 ng input, equivalent to the amount for poor FFPE RNA. This kit is provided with custom baited probes for target enrichment, and the probes can be automatically designed with the e-array tool on the Agilent SureDesign portal starting from any given sequence of the target of interest. In our case, all the full or partial (> 9000 bp) genome sequences, available at the time of probe design, were used as input (GenBank accession numbers NC_005283, MF589987, HQ829973, HQ829972, KU720625, KU720624, and KU720623).

Briefly, the protocol consists in RNA thermal fragmentation, random reverse transcription and second strand cDNA synthesis, adapters ligation, target enrichment with biotynilated baits and streptavidin magnetic beads, index PCR. Final libraries were checked with the Bioanalyzer High Sensitivity DNA kit (Agilent Technologies) and DNA molarity was assessed using the NEBNext Library Quant Kit for Illumina (New England Biolabs, Ipswich, MA, USA).

After a quality check, 14 samples were selected for a first run on an Illumina MiSeq ((Illumina, San Diego, CA, USA) platform with a 2 × 150 bp paired-end protocol, according to the library prep kit guidelines. Due to low library concentration, two samples were sequenced on a separate run, in order to increase the output in terms of raw reads.

Samples 22,497 and 20,673 were processed separately by using a combination of sequence-independent/ single-primer amplification (SISPA) and next generation sequencing as previously described^36^. Library preparation was carried out by using the Nextera XT Library Prep kit (Illumina) according to the manufacturer’s protocol and analyzed on a Illumina NextSeq500 platform using the NextSeq 500/550 Mid Output Reagent Cartridge v2, 300 cycles and standard 150 bp paired-end reads.

### Data analysis and phylogenetics

Raw reads underwent a quality check, trimming reads with quality < Q30 and shorter than 30 bp with cutadapt v1.15^37^. The filtered reads were mapped to the reference sequence (GenBank acc. Num. NC_005283.1) with bwa v0.7.17^38^ and statistics about mean coverage and proportion of DMV reads on the total were retrieved with samtools v1.9–1^39^. A de novo assembly was also performed to investigate whether a single contig could have been retrieved and to obtain information about the non-DMV contigs. Three different softwares were used for the assembly: SPAdes, Trinity and MEGAHIT with default options and a minimum contig length of 700 bp^40–42^. Contigs were merged in a single file and only unique sequences were selected with dedupe.sh and classified using blastn against a local nr database^43,44^. The genome sequences were submitted to NCBI GenBank with accession numbers MN606000–MN606015.

The 16 novel sequences were aligned with Clustal Omega v1.2.4^45^ to the 12 available on GenBank. In details, we chose only the sequences belonging to DMV strain only and not other CeMV strains (e.g. Porpoise morbillivirus). Sequences from cell culture by Jo and colleagues were excluded as well, since they might cause a bias in the phylogeographic analysis. . In order to compare sequence identity, a sliding window analysis was performed with the SimPlot v. 3.5.1 software, with the following settings: window: 200 bp, Step: 20 bp, GapStrip: On, Kimura (2-parameter), T/t: 2.0. Data were plotted using the ggplot2 package within R v3.5.2^46–48^.

The multiple alignment was manipulated with Mesquite v3.6^49^ to create a subset for each viral gene, namely N, PVC, M, F, H, L, and subsequently translated into the respective proteins in order to identify amino acid substitutions based on their biochemical properties (charged, polar, amphipathic, hydrophobic). A substitution was considered when common to all the samples belonging to a given strain: Mediterranean 2006–2008 (including Mexican samples), Mexico-only, NE-Atlantic, North Sea variant (NSV), and Mediterranean 1990–1992.

For phylogeneticsphylogeography, the reference genome NC_005283 was removed because of probable recombination events. The alignments for each gene and for the noncoding sequences were imported in BEAUti v2.6.3. Information about collection site was added as a discrete trait, grouping locations based on the sea of origin in order to reduce the number of variables and to have larger groups^50^. Sea groups were: Adriatic (n = 7), Balearic (n = 2), Mexico (n = 3), North Sea (n = 3), Pelagos Sanctuary (n = 9), and Tyrrhenian without Pelagos Sanctuary (n = 3).

Phylogeographic reconstruction was inferred using BEAST v2.6.3and the packages BEAST_CLASSIC v1.5.0, phylodynamics v1.3.0, and MODEL_SELECTION v1.5.3^51^.

The best nucleotide substitution model was selected for each data partition using bModelTest v1.2.1, returning the GTR as best model for H, M, and PVC genes, GTR + G + I for F, N, and L genes, and JC69 + G + I for the noncoding partition^52–54^. This output was then applied to the dataset for the model selection by path sampling method between the combinations of strict, relaxed exponential and relaxed log-normal molecular clock with different population models (Extended Bayesian Skyline, Bayesian Skyline, Coalescent Constant Population, Birth Death Skyline Serial). Bayes factors are reported in Table S3. The Relaxed log-normal molecular clock with Coalescent Constant Population model was selected based on the Bayes factor. Two separate chains were run for 100,000,000 generations and the outputs were merged with LogCombiner v2.4.8 with a 10% burn-in. Traces of the log files were analyzed with Tracer v1.6.0 and the MCMC runs converged; all the parameters returned an Estimated Sample Size (ESS) > 200.The phylogenetic tree annotated with the location traits was analyzed using FigTree v1.4.4 and the densitree was realized with DensiTree v.2.2.7. To test whether the phylogenetic inference was based on data, we performed the same analysis without using sequence data (sampleFromPrior = "true"). The distributions of the evaluated parameters were different between the runs with and without data, but values of the runs with data were within the range of the priors-only runs, meaning that the priors did not influence the output of the analysis (See Figure S3 for an example). All the BEAST2 MCMC analyses were performed on CIPRES Science Gateway (www.phylo.org). The BEAUti XML files are available as supplementary material.

A phylogenetic tree was also built by MrBayes v. 3.2.6, based on whole data set partitioned by gene and on the single genes and the corresponding nucleotide substitution model, as obtained by bModelTest, was assigned to the *lset* and *prset* parameters^55,56^. The MCMC parallel runs converged and the all the parameters returned an ESS > 200.

## Supplementary information

Supplementary Information.
