# Supplementary material for: Specific capture and whole-genome phylogeography of Dolphin morbillivirus
Source: Sci Rep. 2020 Nov 30;10:20831. doi: 10.1038/s41598-020-77835-z (PMC7704663; doi:10.1038/s41598-020-77835-z)

# *Specific capture and whole-genome phylodynamics of Dolphin morbillivirus*

**F. Cerutti, F. Giorda, C. Grattarola, W. Mignone, C. Beltramo, N. Keck, A. Lorusso, G. Di Francesco, L. Di Renzo, G. Di Guardo, M. Goria, L. Masoero, P.L. Acutis, C. Casalone, S. Peletto**

## Supplementary information

**Table S1. Nucleotide similarity matrix, colored by gradient of similarity. Red: minimum similarity (98.3%); green: maximum similarity (100%).**

| | NC005283_ES_Balearic_1990 | KU720625_USA_Mexico_2011 | KU720624_USA_Mexico_2011 | KU720623_USA_Mexico_2010 | 22497_LE_Adriatic_2017 | 20673_TE_Adriatic_2017 | 3618_RM_Tyrrhenian_2009 | MF589987_SV_Pelagos_2008 | 3908_IM_Pelagos_2015 | 95842_GE_Pelagos_2016 | 6020_TA_Adriatic_2017 | 6023_LE_Adriatic_2017 | 19929_ME_Tyrrhenian_2013 | 26823_IM_Pelagos_2017 | 59728_SS_Pelagos_2011 | 59780_CH_Adriatic_2014 | 78983_SV_Pelagos_2017 | 80729_SA_Pelagos_2011 | 85537_FR_Pelagos_2010 | 85548_FR_Pelagos_2008 | 92300_LE_Adriatic_2016 | MH430941_NL_North_Sea_2011 | MH430940_DE_North_Sea_2007 | MH430939_DK_North_Sea_2016 | MH430938-Bph_IT_Pelagos_2013 | MH430937-156_IT_Tyrrhenian_2010 | MH430935-muc_ES_Balearic_1990 | MH430934-16A_ES_Balearic_1990 |
|---|---|---|---|---|---|---|---|---|---|---|---|---|---|---|---|---|---|---|---|---|---|---|---|---|---|---|---|---|
| NC005283_ES_Balearic_1990 | 100.0% | 98.8% | 98.8% | 98.8% | 98.8% | 98.8% | 99.1% | 99.0% | 99.1% | 99.5% | 99.3% | 98.8% | 99.0% | 98.9% | 98.9% | 99.1% | 98.8% | 99.0% | 99.0% | 99.0% | 98.8% | 98.4% | 98.6% | 98.8% | 98.9% | 98.9% | 99.5% | 99.5% |
| KU720625_USA_Mexico_2011 | 98.8% | 100.0% | 99.9% | 99.8% | 99.1% | 99.0% | 99.5% | 99.5% | 99.5% | 99.5% | 99.6% | 99.0% | 99.4% | 99.1% | 99.4% | 99.4% | 99.1% | 99.4% | 99.5% | 99.5% | 99.1% | 98.3% | 98.5% | 99.1% | 99.4% | 99.4% | 99.3% | 99.2% |
| KU720624_USA_Mexico_2011 | 98.8% | 99.9% | 100.0% | 99.8% | 99.0% | 99.0% | 99.5% | 99.5% | 99.5% | 99.5% | 99.6% | 99.0% | 99.4% | 99.1% | 99.4% | 99.3% | 99.1% | 99.4% | 99.5% | 99.5% | 99.0% | 98.3% | 98.5% | 99.0% | 99.4% | 99.4% | 99.2% | 99.2% |
| KU720623_USA_Mexico_2010 | 98.8% | 99.8% | 99.8% | 100.0% | 99.1% | 99.1% | 99.5% | 99.5% | 99.5% | 99.6% | 99.7% | 99.1% | 99.5% | 99.2% | 99.5% | 99.4% | 99.1% | 99.5% | 99.5% | 99.5% | 99.1% | 98.3% | 98.5% | 99.1% | 99.5% | 99.4% | 99.3% | 99.3% |
| 22497_LE_Adriatic_2017 | 98.8% | 99.1% | 99.0% | 99.1% | 100.0% | 99.9% | 99.3% | 99.3% | 99.5% | 100.0% | 100.0% | 99.9% | 99.2% | 99.9% | 99.2% | 100.0% | 99.8% | 99.2% | 99.3% | 99.3% | 99.9% | 98.3% | 98.5% | 99.6% | 99.2% | 99.2% | 99.2% | 99.2% |
| 20673_TE_Adriatic_2017 | 98.8% | 99.0% | 99.0% | 99.1% | 99.9% | 100.0% | 99.3% | 99.3% | 99.5% | 100.0% | 100.0% | 100.0% | 99.2% | 99.9% | 99.2% | 100.0% | 99.8% | 99.2% | 99.3% | 99.3% | 100.0% | 98.3% | 98.5% | 99.6% | 99.2% | 99.1% | 99.2% | 99.2% |
| 3618_RM_Tyrrhenian_2009 | 99.1% | 99.5% | 99.5% | 99.5% | 99.3% | 99.3% | 100.0% | 99.9% | 99.9% | 99.8% | 99.9% | 99.3% | 99.9% | 99.4% | 99.9% | 99.7% | 99.4% | 99.9% | 99.9% | 99.9% | 99.3% | 98.6% | 98.8% | 99.3% | 99.9% | 99.9% | 99.5% | 99.5% |
| MF589987_SV_Pelagos_2008 | 99.0% | 99.5% | 99.5% | 99.5% | 99.3% | 99.3% | 99.9% | 100.0% | 99.8% | 99.8% | 100.0% | 99.3% | 99.8% | 99.3% | 99.8% | 99.6% | 99.3% | 99.8% | 99.9% | 99.9% | 99.3% | 98.5% | 98.7% | 99.3% | 99.8% | 99.8% | 99.4% | 99.4% |
| 3908_IM_Pelagos_2015 | 99.1% | 99.5% | 99.5% | 99.5% | 99.5% | 99.5% | 99.9% | 99.8% | 100.0% | 99.8% | 99.9% | 99.5% | 100.0% | 99.5% | 99.9% | 99.7% | 99.5% | 100.0% | 99.8% | 99.9% | 99.5% | 98.5% | 98.8% | 99.4% | 99.8% | 99.8% | 99.5% | 99.5% |
| 95842_GE_Pelagos_2016 | 99.5% | 99.5% | 99.5% | 99.6% | 100.0% | 100.0% | 99.8% | 99.8% | 99.8% | 100.0% | 100.0% | 100.0% | 99.7% | 100.0% | 99.7% | 100.0% | 99.9% | 99.7% | 99.7% | 99.8% | 100.0% | 98.9% | 99.1% | 99.8% | 99.6% | 99.6% | 100.0% | 99.9% |
| 6020_TA_Adriatic_2017 | 99.3% | 99.6% | 99.6% | 99.7% | 100.0% | 100.0% | 99.9% | 100.0% | 99.9% | 100.0% | 100.0% | 100.0% | 99.9% | 100.0% | 99.9% | 100.0% | 99.9% | 99.9% | 100.0% | 100.0% | 100.0% | 98.8% | 98.9% | 99.8% | 99.8% | 99.8% | 99.7% | 99.7% |
| 6023_LE_Adriatic_2017 | 98.8% | 99.0% | 99.0% | 99.1% | 99.9% | 100.0% | 99.3% | 99.3% | 99.5% | 100.0% | 100.0% | 100.0% | 99.2% | 99.9% | 99.2% | 100.0% | 99.8% | 99.2% | 99.3% | 99.3% | 100.0% | 98.3% | 98.5% | 99.6% | 99.2% | 99.2% | 99.2% | 99.2% |
| 19929_ME_Tyrrhenian_2013 | 99.0% | 99.4% | 99.4% | 99.5% | 99.2% | 99.2% | 99.9% | 99.8% | 100.0% | 99.7% | 99.9% | 99.2% | 100.0% | 99.3% | 99.9% | 99.6% | 99.2% | 100.0% | 99.8% | 99.8% | 99.2% | 98.5% | 98.7% | 99.2% | 99.8% | 99.8% | 99.4% | 99.4% |
| 26823_IM_Pelagos_2017 | 98.9% | 99.1% | 99.1% | 99.2% | 99.9% | 99.9% | 99.4% | 99.3% | 99.5% | 100.0% | 100.0% | 99.9% | 99.3% | 100.0% | 99.3% | 100.0% | 100.0% | 99.3% | 99.3% | 99.4% | 99.9% | 98.4% | 98.5% | 99.7% | 99.3% | 99.2% | 99.3% | 99.3% |
| 59728_SS_Pelagos_2011 | 98.9% | 99.4% | 99.4% | 99.5% | 99.2% | 99.2% | 99.9% | 99.8% | 99.9% | 99.7% | 99.9% | 99.2% | 99.9% | 99.3% | 100.0% | 99.6% | 99.2% | 99.9% | 99.8% | 99.8% | 99.2% | 98.4% | 98.6% | 99.2% | 99.9% | 99.9% | 99.4% | 99.3% |
| 59780_CH_Adriatic_2014 | 99.1% | 99.4% | 99.3% | 99.4% | 100.0% | 100.0% | 99.7% | 99.6% | 99.7% | 100.0% | 100.0% | 100.0% | 99.6% | 100.0% | 99.6% | 100.0% | 99.9% | 99.6% | 99.6% | 99.7% | 100.0% | 98.6% | 98.8% | 99.7% | 99.5% | 99.5% | 99.5% | 99.5% |
| 78983_SV_Pelagos_2017 | 98.8% | 99.1% | 99.1% | 99.1% | 99.8% | 99.8% | 99.4% | 99.3% | 99.5% | 99.9% | 99.9% | 99.8% | 99.2% | 100.0% | 99.2% | 99.9% | 100.0% | 99.2% | 99.3% | 99.3% | 99.9% | 98.3% | 98.5% | 99.6% | 99.2% | 99.2% | 99.2% | 99.2% |
| 80729_SA_Pelagos_2011 | 99.0% | 99.4% | 99.4% | 99.5% | 99.2% | 99.2% | 99.9% | 99.8% | 100.0% | 99.7% | 99.9% | 99.2% | 100.0% | 99.3% | 99.9% | 99.6% | 99.2% | 100.0% | 99.8% | 99.8% | 99.2% | 98.4% | 98.7% | 99.2% | 99.8% | 99.8% | 99.4% | 99.3% |
| 85537_FR_Pelagos_2010 | 99.0% | 99.5% | 99.5% | 99.5% | 99.3% | 99.3% | 99.9% | 99.9% | 99.8% | 99.7% | 100.0% | 99.3% | 99.8% | 99.3% | 99.8% | 99.6% | 99.3% | 99.8% | 100.0% | 99.9% | 99.3% | 98.5% | 98.7% | 99.2% | 99.8% | 99.7% | 99.4% | 99.4% |
| 85548_FR_Pelagos_2008 | 99.0% | 99.5% | 99.5% | 99.5% | 99.3% | 99.3% | 99.9% | 99.9% | 99.9% | 99.8% | 100.0% | 99.3% | 99.8% | 99.4% | 99.8% | 99.7% | 99.3% | 99.8% | 99.9% | 100.0% | 99.3% | 98.5% | 98.7% | 99.3% | 99.8% | 99.8% | 99.5% | 99.4% |
| 92300_LE_Adriatic_2016 | 98.8% | 99.1% | 99.0% | 99.1% | 99.9% | 100.0% | 99.3% | 99.3% | 99.5% | 100.0% | 100.0% | 100.0% | 99.2% | 99.9% | 99.2% | 100.0% | 99.9% | 99.2% | 99.3% | 99.3% | 100.0% | 98.3% | 98.5% | 99.6% | 99.2% | 99.2% | 99.2% | 99.2% |
| MH430941_NL_North_Sea_2011 | 98.4% | 98.3% | 98.3% | 98.3% | 98.3% | 98.3% | 98.6% | 98.5% | 98.5% | 98.9% | 98.8% | 98.3% | 98.5% | 98.4% | 98.4% | 98.6% | 98.3% | 98.4% | 98.5% | 98.5% | 98.3% | 100.0% | 99.6% | 98.4% | 98.4% | 98.4% | 98.8% | 98.8% |
| MH430940_DE_North_Sea_2007 | 98.6% | 98.5% | 98.5% | 98.5% | 98.5% | 98.5% | 98.8% | 98.7% | 98.8% | 99.1% | 98.9% | 98.5% | 98.7% | 98.5% | 98.6% | 98.8% | 98.5% | 98.7% | 98.7% | 98.7% | 98.5% | 99.6% | 100.0% | 98.5% | 98.7% | 98.6% | 99.1% | 99.0% |
| MH430939_DK_North_Sea_2016 | 98.8% | 99.1% | 99.0% | 99.1% | 99.6% | 99.6% | 99.3% | 99.3% | 99.4% | 99.8% | 99.8% | 99.6% | 99.2% | 99.7% | 99.2% | 99.7% | 99.6% | 99.2% | 99.2% | 99.3% | 99.6% | 98.4% | 98.5% | 100.0% | 99.2% | 99.2% | 99.3% | 99.2% |
| MH430938-Bph_IT_Pelagos_2013 | 98.9% | 99.4% | 99.4% | 99.5% | 99.2% | 99.2% | 99.9% | 99.8% | 99.8% | 99.6% | 99.8% | 99.2% | 99.8% | 99.3% | 99.9% | 99.5% | 99.2% | 99.8% | 99.8% | 99.8% | 99.2% | 98.4% | 98.7% | 99.2% | 100.0% | 100.0% | 99.4% | 99.4% |
| MH430937-156_IT_Tyrrhenian_2010 | 98.9% | 99.4% | 99.4% | 99.4% | 99.2% | 99.1% | 99.9% | 99.8% | 99.8% | 99.6% | 99.8% | 99.2% | 99.8% | 99.2% | 99.9% | 99.5% | 99.2% | 99.8% | 99.7% | 99.8% | 99.2% | 98.4% | 98.6% | 99.2% | 100.0% | 100.0% | 99.4% | 99.3% |
| MH430935-muc_ES_Balearic_1990 | 99.5% | 99.3% | 99.2% | 99.3% | 99.2% | 99.2% | 99.5% | 99.4% | 99.5% | 100.0% | 99.7% | 99.2% | 99.4% | 99.3% | 99.4% | 99.5% | 99.2% | 99.4% | 99.4% | 99.5% | 99.2% | 98.8% | 99.1% | 99.3% | 99.4% | 99.4% | 100.0% | 99.9% |
| MH430934-16A_ES_Balearic_1990 | 99.5% | 99.2% | 99.2% | 99.3% | 99.2% | 99.2% | 99.5% | 99.4% | 99.5% | 99.9% | 99.7% | 99.2% | 99.4% | 99.3% | 99.3% | 99.5% | 99.2% | 99.3% | 99.4% | 99.4% | 99.2% | 98.8% | 99.0% | 99.2% | 99.4% | 99.3% | 99.9% | 100.0% |

**Table S2. Recombination analysis as reported by RDP4.**

| Recombination Event Number | Breakpoint Positions relative to NC_005283: Begin | Breakpoint Positions relative to NC_005283: End | Recombinant Sequence(s) | Minor Parental Sequence(s) | Major Parental Sequence(s) | Detection Methods: RDP | GENECONV | Bootscan | Maxchi | Chimaera | SiSscan | 3Seq |
|---|---|---|---|---|---|---|---|---|---|---|---|---|
| 1 | 90* | 10440 | ^NC_005283_ES_Balearic_1990 | MH430934-16A_ES_Balearic_1990 | Unknown (MH430937-156_IT_Tyrrhenian_2010) | 2.94E-06 | 4.45E-07 | 3.01E-05 | 5.19E-09 | 1.20E-11 | 4.11E-10 | 6.22E-14 |
| 1 | | | | MH430935-muc_ES_Balearic_1990 | Unknown(KU720625_USA_Mexico_2011) | | | | | | | |
| 1 | | | | | Unknown(KU720624_USA_Mexico_2011) | | | | | | | |
| 1 | | | | | Unknown(KU720623_USA_Mexico_2010) | | | | | | | |
| 1 | | | | | Unknown(MN606000_22497_TE_Adriatic_2017) | | | | | | | |
| 1 | | | | | Unknown(MN606001_20673_TE_Adriatic_2017) | | | | | | | |
| 1 | | | | | Unknown(MN606002_3618_RM_Tyrrhenian_2009) | | | | | | | |
| 1 | | | | | Unknown(MF589987_SV_Pelagos_2008) | | | | | | | |
| 1 | | | | | Unknown(MN606003_3908_IM_Pelagos_2015) | | | | | | | |
| 1 | | | | | Unknown(MN606006_6023_LE_Adriatic_2017) | | | | | | | |
| 1 | | | | | Unknown(MN606007_19929_ME_Tyrrhenian_2013) | | | | | | | |
| 1 | | | | | Unknown(MN606008_26823_IM_Pelagos_2017) | | | | | | | |
| 1 | | | | | Unknown(MN606010_59728_SS_Pelagos_2011) | | | | | | | |
| 1 | | | | | Unknown(MN606011_78983_SV_Pelagos_2017) | | | | | | | |
| 1 | | | | | Unknown(MN606012_80729_SA_Pelagos_2011) | | | | | | | |
| 1 | | | | | Unknown(MN606013_85537_FR_Pelagos_2010) | | | | | | | |
| 1 | | | | | Unknown(MN606014_85548_FR_Pelagos_2008) | | | | | | | |
| 1 | | | | | Unknown(MN606015_92300_LE_Adriatic_2016) | | | | | | | |
| 1 | | | | | Unknown(MH430938-Bph_IT_Pelagos_2013) | | | | | | | |
| 2 | 12091* | 12459 | NC_005283_ES_Balearic_1990 | Unknown (MH430940_DE_North_Sea_2007) | MN606002_3618_RM_Tyrrhenian_2009 | 1.58E-03 | 1.61E-03 | NS | NS | NS | NS | NS |
| 2 | | | | Unknown(MH430941_NL_North_Sea_2011) | | | | | | | | |
| 3 | 11566 | 14929* | ^MH430937-156_IT_Tyrrhenian_2010 | KU720623_USA_Mexico_2010 | MN606002_3618_RM_Tyrrhenian_2009 | NS | 0.009072 | NS | NS | NS | 2.95E-06 | NS |
| 4 | 5224 | 5342 | MH430939_DK_North_Sea_2016 | Unknown (KU720624_USA_Mexico_2011) | MN606000_22497_PE_Adria c_2017 | NS | 0.02772 | 4.62E-03 | NS | NS | NS | NS |
| 4 | | | | Unknown(KU720625_USA_Mexico_2011) | MN606011_78983_SV_Pelagos_2017 | | | | | | | |
| 4 | | | | Unknown(KU720623_USA_Mexico_2010) | | | | | | | | |

Table Key:

~ = It is possible that this apparent recombination signal could have been caused by an evolutionary process other than recombination.

* = The actual breakpoint position is undetermined (it was most likely overprinted by a subsequent recombination event).

^ = The recombinant sequence may have been misidentified (one of the identified parents might be the recombinant)

Minor Parent = Parent contributing the smaller fraction of sequence.

Major Parent = Parent contributing the larger fraction of sequence.

Unknown = Only one parent and a recombinant need be in the alignment for a recombination event to be detectable. The sequence listed as unknown was used to infer the existance of a missing parental sequence.

NS = No significant P-value was recorded for this recombination event using this method.

**Table S3. Bayes factor for the models tested with path sampling for the BEAST analysis.** Models are sorted based on the marginal likelihood, and the Bayes factor between each model and the best model is reported. The sum(ESS) is reported for each model as well.

| Model | Marginal likelihood | sum(ESS) | Bayes factor vs best model |
|---|---|---|---|
| **Relaxed log Coalescent Constant Population** | **-26161.02** | 2120.9 | 0.00 |
| Relaxed exponential Extended Bayesian Skyline | -26163.99 | 2486.7 | -2.97 |
| Relaxed log Bayesian Skyline | -26165.15 | 2563.9 | -4.13 |
| Relaxed exponential Coalescent Constant Population | -26165.64 | 2066.2 | -4.62 |
| Relaxed exponential Birth Death Skyline Serial | -26166.40 | 2178.9 | -5.38 |
| Relaxed exponential Bayesian Skyline | -26166.92 | 2174.6 | -5.90 |
| Relaxed log Extended Bayesian Skyline | -26167.06 | 2547.1 | -6.04 |
| Relaxed log Birth Death Skyline Serial | -26175.01 | 1830.3 | -13.99 |
| Strict Extended Bayesian Skyline | -26177.94 | 2958.1 | -16.92 |
| Strict Bayesian Skyline | -26178.10 | 2688.2 | -17.09 |
| Strict Coalescent Constant Population | -26178.81 | 2613.6 | -17.79 |
| Strict Birth Death Skyline Serial | -26189.85 | 2593.6 | -28.83 |

**Figure S1. Sliding window analysis using the DMV reference genome sequence NC_005283 as query. Window size: 200 bp, Step: 20 bp.** Dashed lines mark the region, with a length spanning between 12,000 and 12,500 bp, where the reference viral genome shows a higher diversity.

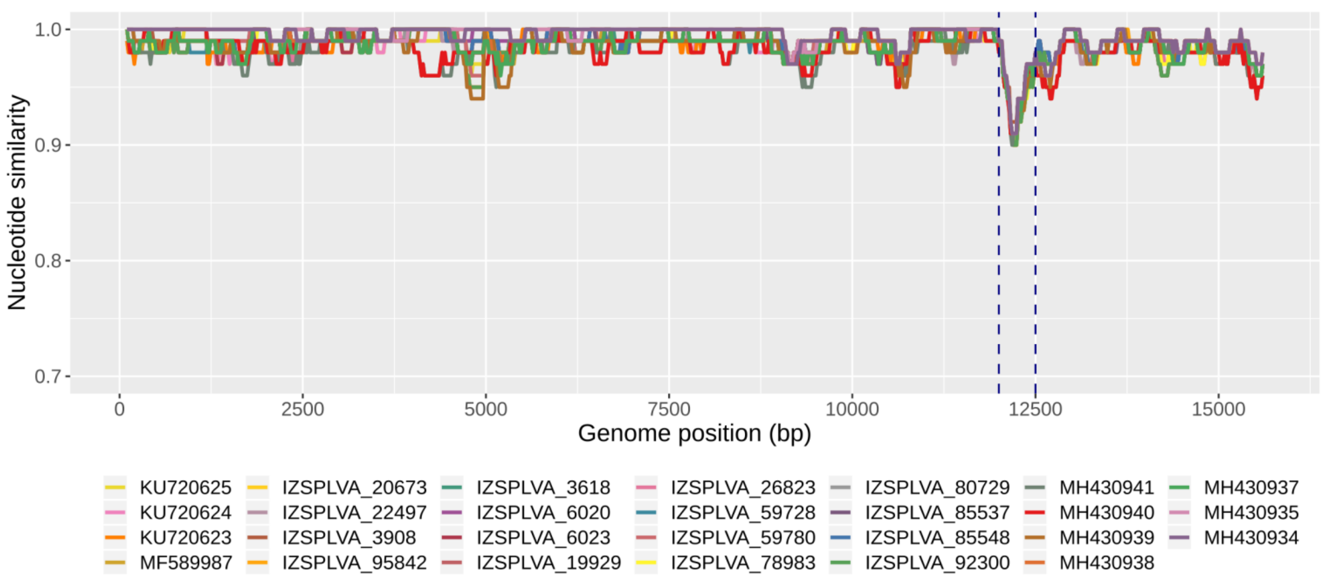

**Figure S2. DensiTree from the phylogeographic analysis with BEAST2.** Branches are colored based on the Sea.

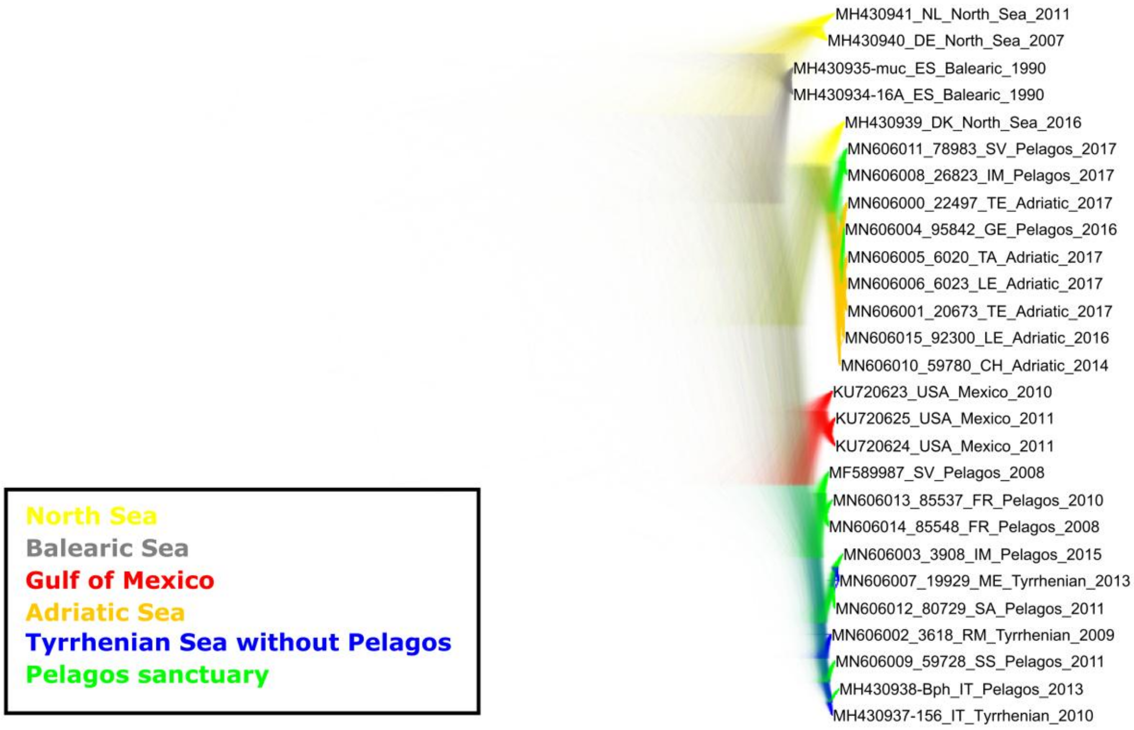

**Figure S3. Comparison of posterior (black) and prior-only (blue) distributions of some parameters.**

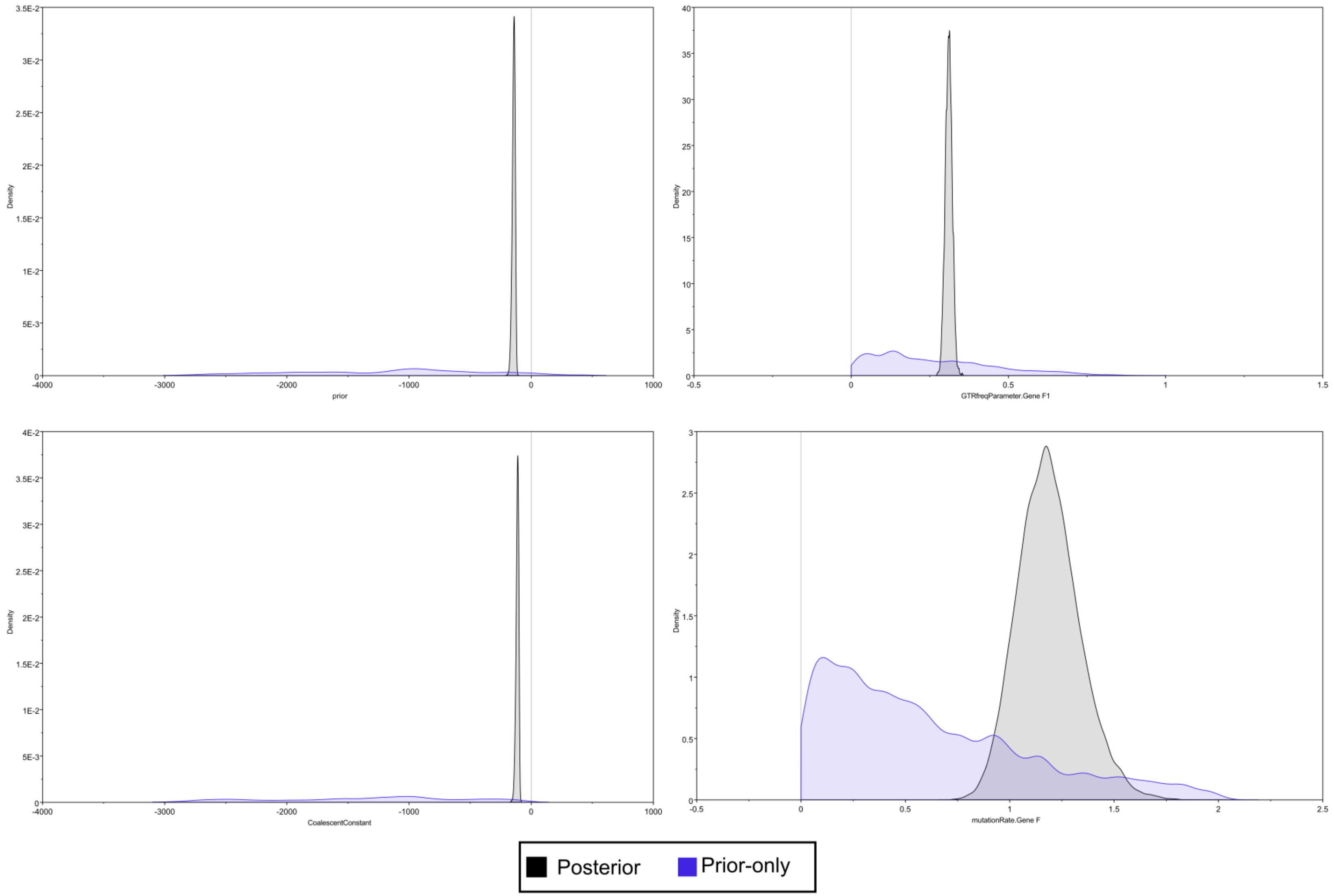

Supplement: Supplementary file 1 — Supplementary Information. [file 41598_2020_77835_MOESM1_ESM.pdf]
